# Nicotinamide alone accelerates the conversion of mouse embryonic stem cells into mature neuronal populations

**DOI:** 10.1371/journal.pone.0183358

**Published:** 2017-08-17

**Authors:** Síle M. Griffin, Mark R. Pickard, Rowan P. Orme, Clive P. Hawkins, Adrian C. Williams, Rosemary A. Fricker

**Affiliations:** 1 Keele Medical School and Institute for Science and Technology in Medicine, Keele University, Keele, Staffordshire, England, United Kingdom; 2 Chester Centre for Stress Research, Institute of Medicine, University of Chester, Chester, United Kingdom; 3 Department of Neurology, University Hospital of North Staffordshire, Stoke-on-Trent, Staffordshire, England, United Kingdom; 4 Department of Neurosciences, University of Birmingham, Birmingham, United Kingdom; University of Texas at Austin Dell Medical School, UNITED STATES

## Abstract

**Introduction:**

Vitamin B_3_ has been shown to play an important role during embryogenesis. Specifically, there is growing evidence that nicotinamide, the biologically active form of vitamin B_3_, plays a critical role as a morphogen in the differentiation of stem cells to mature cell phenotypes, including those of the central nervous system (CNS). Detailed knowledge of the action of small molecules during neuronal differentiation is not only critical for uncovering mechanisms underlying lineage-specification, but also to establish more effective differentiation protocols to obtain clinically relevant cells for regenerative therapies for neurodegenerative conditions such as Huntington’s disease (HD). Thus, this study aimed to investigate the potential of nicotinamide to promote the conversion of stem cells to mature CNS neurons.

**Methods:**

Nicotinamide was applied to differentiating mouse embryonic stem cells (mESC; *Sox1*GFP knock-in 46C cell line) during their conversion towards a neural fate. Cells were assessed for changes in their proliferation, differentiation and maturation; using immunocytochemistry and morphometric analysis methods.

**Results:**

Results presented indicate that 10 mM nicotinamide, when added at the initial stages of differentiation, promoted accelerated progression of ESCs to a neural lineage in adherent monolayer cultures. By 14 days *in vitro* (DIV), early exposure to nicotinamide was shown to increase the numbers of differentiated βIII-tubulin-positive neurons. Nicotinamide decreased the proportion of pluripotent stem cells, concomitantly increasing numbers of neural progenitors at 4 DIV. These progenitors then underwent rapid conversion to neurons, observed by a reduction in *Sox 1* expression and decreased numbers of neural progenitors in the cultures at 14 DIV. Furthermore, GABAergic neurons generated in the presence of nicotinamide showed increased maturity and complexity of neurites at 14 DIV. Therefore, addition of nicotinamide alone caused an accelerated passage of pluripotent cells through lineage specification and further to non-dividing mature neurons.

**Conclusions:**

Our results show that, within an optimal dose range, nicotinamide is able to singly and selectively direct the conversion of embryonic stem cells to mature neurons, and therefore may be a critical factor for normal brain development, thus supporting previous evidence of the fundamental role of vitamins and their metabolites during early CNS development. In addition, nicotinamide may offer a simple effective supplement to enhance the conversion of stem cells to clinically relevant neurons.

## Introduction

Human pluripotent stem cells are powerful contenders to alleviate a myriad of debilitating brain-related degenerative disorders, directly as cell replacement therapies [1], or indirectly through the development of *in vitro* models for the study of mechanisms underlying human neural development, disease modelling, drug screening and neuroprotection assays [2]. However, clinical translations of stem cell candidates, such as embryonic stem cells (ESCs), can only commence once important challenges have been adequately resolved and protocols are improved to restrict stem cell proliferation linked to tumour formation, and to promote differentiation of ESCs to higher and purer yields of desired cell phenotypes [3]. Furthermore, understanding the mechanisms governing neural progenitor differentiation, neuronal fate specification, maturation and survival of developing stem cell-derived neurons is crucial to advance cutting-edge research in translational medicine.

Differentiation of neural progenitors into postmitotic neurons requires precise coordination of inductive signals required to inhibit self-renewal combined with signals that drive the programme of terminal differentiation. Thus, knowledge of the effects and timing of inductive molecules is fundamental for advancing prospective therapies to generate stem cell-derived neuronal populations. In this regard, vitamins are well known to play crucial roles during early neuronal development in embryogenesis, and a number of studies have shown that key signalling proteins for vitamins are being expressed at the correct time and place to directly influence neural development [4–6]. The biologically active metabolites of vitamin C (ascorbic acid), vitamin D_3_ (calcitriol) and vitamin A (retinoic acid) are frequently included in differentiation strategies to enhance the *in vitro* derivation of specific postmitotic subtypes from stem cells or neural progenitors [7–17].

The identification of nicotinamide as a novel morphogen pointed to a critical, early role of the vitamin B_3_ metabolite during the process of differentiation to influence cell fate specification [18]. The early developmental role of nicotinamide is further supported by historical examples of neurodegenerative pathology observed in motor neurons as a result of nicotinamide deficiency [19]. Further, in human Pellagra, nicotinamide and tryptophan deficiency leads to range of symptoms including dermatitis, diarrhoea, dementia, depression and other features of neurological disorders including Parkinsonism [20]. The ability of nicotinamide as a differentiation agent to induce postmitotic neural phenotypes has been reported in various stem cells *in vitro* [12,15–17]. However, there is a paucity of information currently on the potential of nicotinamide to drive ESC differentiation into neural and neuronal populations.

The present study describes a thorough investigation as to whether nicotinamide could influence the conversion of mESCs undergoing neural differentiation to mature neurons, using an adherent serum-free and factor-free monolayer differentiation protocol [21]. A mESC cell line engineered to express a reporter of neural specification, *Sox1*GFP, was employed here, to facilitate direct visualisation of the neural progenitor state in living cells with fluorescence microscopy [21]. Thus, this advantageous feature of the *Sox1*GFP line allowed, for the first time, a detailed investigation of the effects of nicotinamide, on ESC-derived cell populations at any point during monolayer differentiation. Novel findings show that nicotinamide functions as a potent signalling factor in brain development, and in a definable dosage range and exposure time, accelerates the production and maturation of neurons.

## Results

### Nicotinamide accelerated neural lineage commitment of *Sox1*GFP mESCs

**Fig 1:**
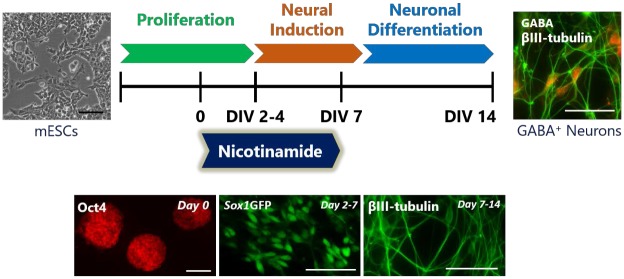
Schematic diagram of time-course analysis of neural and neuronal differentiation in the presence of nicotinamide. Undifferentiated cells were cultured as monolayers in N2B27 medium for different durations to investigate whether nicotinamide would influence the conversion of Oct4^+^undifferentiated stem cells (scale bar = 100 μm) to *Sox1*GFP neural progenitors (scale bar = 50 μm) and thus drive differentiation toward βIII-tubulin^+^ neurons (scale bar = 50 μm). The potential of nicotinamide was assessed using immunocytochemistry at different culture periods from days 2–14.

**Fig 2:**
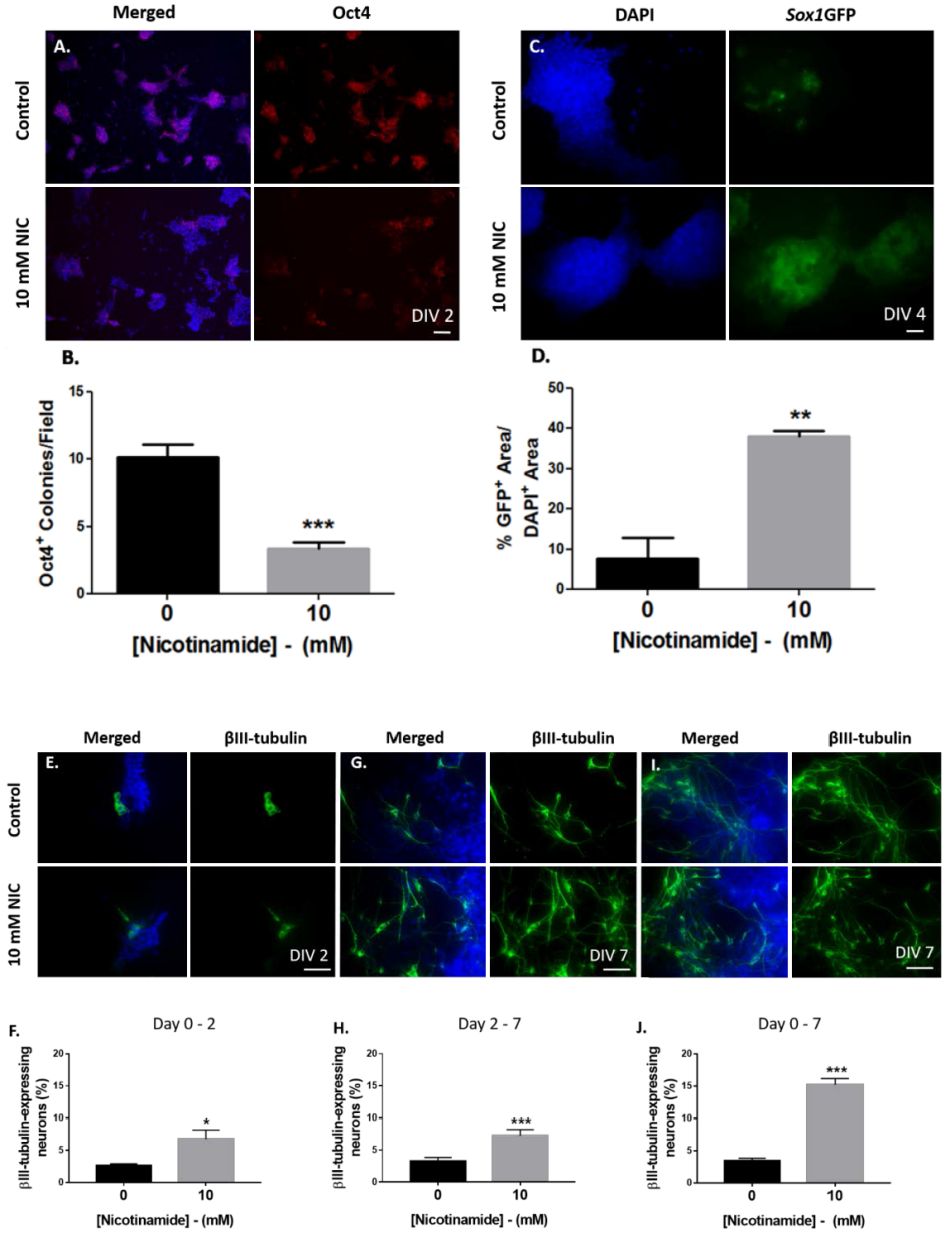
Nicotinamide accelerated neural specification of *Sox1*GFP mESCs. Nicotinamide treatment significantly decreased Oct4^+^ pluripotent cells (A-B) and concomitantly increased GFP^+^ cells (C-D) at day 4, suggesting enhanced neural lineage commitment. The effect of nicotinamide on immature neuron expression was assessed during monolayer culture periods; (E-F) day 0–2, (G-H) day 2–7 and (I-J) day 0–7. Scale bar 100 μm applies to all low magnification images. ***p<0.001,**p<0.01, *p<0.05.

Adherent monolayer cultures supplemented with nicotinamide at day 0 to day 2 and day 2 to day 7 were analysed for the expression of markers of undifferentiated ESCs, early neural differentiation and early neuronal development (Fig 1). To investigate whether nicotinamide could promote mESC entry to the neural lineage in the monolayer culture system, changes in the ESC marker protein, Oct4, were assessed. Nicotinamide added to undifferentiated *Sox1*GFP knock-in reporter mESCs at the onset of differentiation (day 0) caused a significant reduction in the number of Oct4-expressing colonies per field by day 2 (unpaired t test, t = 6.0; *p<*0.001; 3.3 ± 0.5in nicotinamide-treated vs. 10.2 ± 1.0 in controls; Fig 2(A) and 2(B)).

In accordance with other reports, there was a highly reproducible up-regulation of native GFP expression in control cultures, driven by expression of the *Sox1* gene by day 3–4 of monolayer differentiation [22,23]. Therefore, GFP expression was assessed at day 4, in cultures treated with nicotinamide on days 0–2. The percentage of native GFP-labelled regions within DAPI^+^ colonies was significantly enhanced in nicotinamide treated cultures by day 4, compared to controls (unpaired t test, t = 5.6; p<0.01; 38.1 ± 1.4% in nicotinamide treated vs. 7.6 ± 5.2% in control groups; Fig 2(C) and 2(D)).

### Early application of nicotinamide induces a more potent effect on neuronal differentiation

To establish the exact time-window of ESC differentiation where nicotinamide predominantly acts on cells to increase neuronal production, adherent monolayer cultures supplemented with nicotinamide at day 0 to 2, day 2 to day 7 and day 0 to day 7 were also analysed for the expression of βIII-tubulin marker. Consistent with results highlighting a rapid decline in the ESC state and accelerated emergence of GFP^+^ progenitor cells, the expression of βIII-tubulin was also up-regulated in nicotinamide-treated cultures as early as day 7 (the neural induction stage) in this monolayer system. Nicotinamide administered from day 0 to 2 significantly promoted the generation of immature neuronal populations (unpaired t test, t = 2.9; p<0.05; 6.8 ± 1.4% vs. 2.6 ± 0.3% in untreated conditions; Fig 2(E) and 2(F)). Therefore, these data indicate that the processes of neural specification and neuronal differentiation were accelerated, under the driving force of nicotinamide applied at the initial stages of ESC differentiation.

Interestingly, addition of nicotinamide from day 0 of monolayer differentiation elicited a more potent effect on neuronal differentiation by day 7. Monolayer cultures supplemented with nicotinamide treatment from day 0 showed significantly enhanced yields of βIII-tubulin^+^ neurons (unpaired t test, t = 11.6; p<0.001; 15.2 ± 1.0% in day 0–7 treated vs. 3.5 ± 0.4% in control groups; Fig 2(I) and 2(J)), in contrast to nicotinamide treatment between days 2 to 7,which showed less effect (unpaired t test, t = 3.7; p<0.001; 7.3 ± 1.0% vs. 3.3 ± 0.6% in control groups; Fig 2(G) and 2(H)).

Thus, given the direct effect of 10 mM nicotinamide at the early developmental stages of ESC differentiation (i.e. day 0), the following experiments focused on monolayer cultures treated with 10 mM nicotinamide at the initial stages of differentiation (i.e. day 0–7), and the cells were further differentiated up to a total of 14 days.

### Stem cell pluripotency was down-regulated when treated with nicotinamide

**Fig 3:**
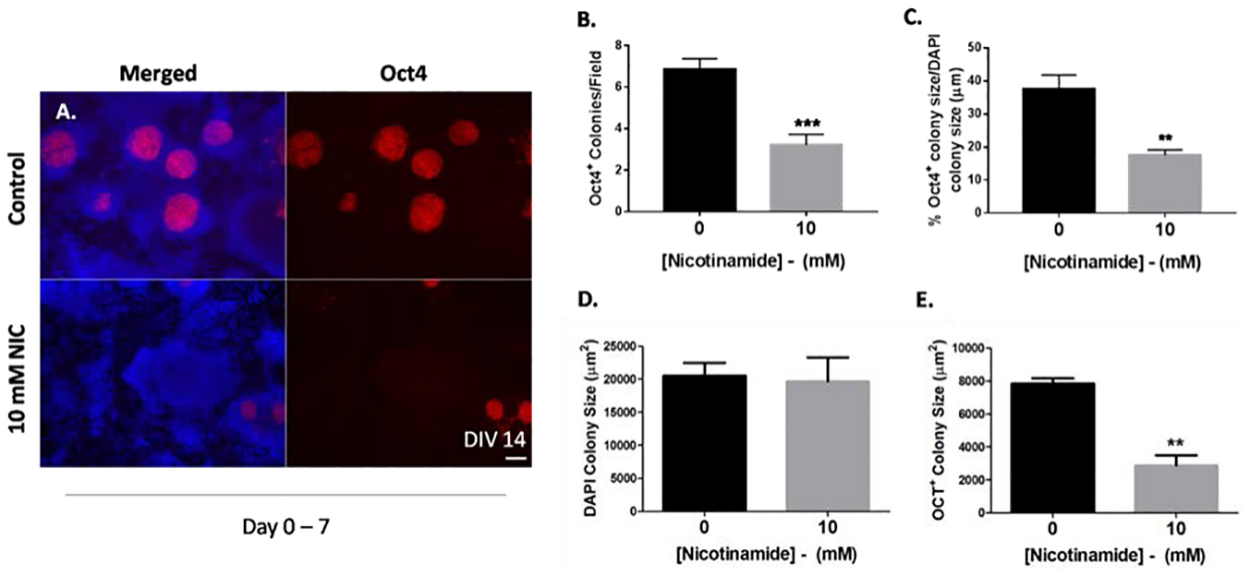
Early nicotinamide treatment accelerates stem cell differentiation. (A-B)Nicotinamide-treated cultures (10 mM added from day 0–7) exhibited significantly lower numbers of Oct4^+^ colonies at day 14. (C) Oct4^+^ colonies were smaller in area within DAPI^+^ colonies in cultures treated with nicotinamide. (D) DAPI colonies were similar in area (μm^2^) across all treatment conditions. (E) Areas of Oct4^+^ colonies were markedly reduced in cultures treated with 10 mM nicotinamide, suggestive of accelerated stem cell differentiation. Scale bar 100 μm applies to all low magnification images. **p<0.01; ***p<0.001.

The number of undifferentiated stem cell colonies per field, (determined by colonies expressing the stem cell marker Oct4 at day 14), was reduced by half when 10 mM nicotinamide was added to cultures at the early phase of neural differentiation (i.e. days 0–7;unpaired t test, t = 5.1; p<0.001; 3.2 ± 0.5 vs. 6.9 ± 0.5 in controls; Fig 3(A) and 3(B)). Therefore, these novel findings indicate a beneficial role for nicotinamide to reduce the numbers of dividing cells in adherent monolayer cultures by day 14.

We explored whether nicotinamide could impact on the phenotype of the remaining undifferentiated colonies at day 14. The loss of the pluripotency regulator Oct4 was examined by measuring colony area size (μm^2^) using Image-J software. Pluripotency was assessed within individual DAPI-labelled colonies as the proportion of cells expressing the Oct4 protein; i.e. a ratio of the region of red fluorescence (Oct4^+^) compared to blue fluorescence (DAPI^+^) within a fixed area. Nicotinamide significantly decreased the size of Oct4-labelled colonies in cultures treated between days 0 and 7. The percentage of Oct4-labelled areas within DAPI^+^ colonies was significantly smaller in 10 mM nicotinamide treated cultures (unpaired t test, t = 3.6; p<0.01; 17.5 ± 1.7% vs. 37.7 ± 4.2% in control conditions; Fig 3(C)). Cells treated between days 7 and 14 did not show any significant differences in Oct4 expression *(data not shown)*.The mean area of DAPI^+^ and OCT4^+^colonies (μm^2^) was calculated for cultures treated at the early stages differentiation (days 0–7) versus controls. DAPI-labelled colonies remained constant in size (unpaired t test, t = 0.2; n.s.; 19680 μm^2^ ± 3625 in cultures with 10 mM nicotinamide vs. 20524 μm^2^ ± 1966 in control conditions; Fig 3(D)). In contrast, 10 mM nicotinamide induced a rapid loss of pluripotency as evidenced by a significant reduction in Oct4^+^ colony size (unpaired t test, t = 7.1; p<0.01; 2877 μm^2^ ± 625.2 vs. 7862 μm^2^ ± 317.1 in controls; Fig 3(E)).

In summary, nicotinamide promoted earlier differentiation of mESCs from pluripotent stem cells to a more mature developmental state, as evidenced by a decrease in Oct4 expression following early nicotinamide exposure. Therefore, early supplementation of differentiating ESC cultures with nicotinamide not only aids the elimination of potential tumorigenic cells from cultures, but also promotes an accelerated loss of pluripotency, thereby speeding up cell-cycle exit from the ESC state.

### Nicotinamide treatment decreases proliferation and accelerates differentiation of progenitor cells towards a neuronal fate

**Fig 4:**
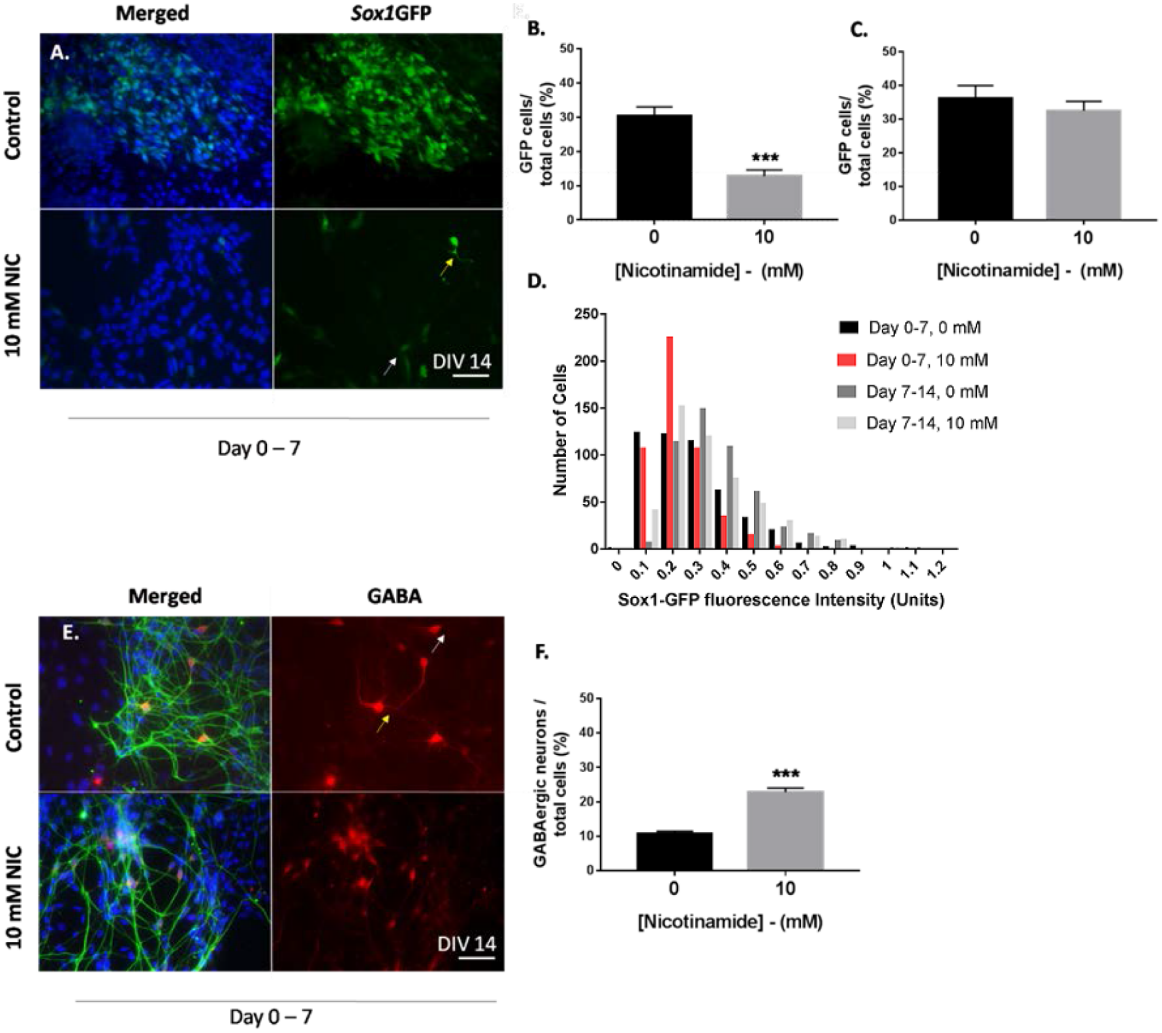
Early nicotinamide administration accelerates the conversion of neural progenitor populations to mature cell types. (A-B) Early nicotinamide treatment (days 0–7) induced a significant decrease in the *Sox1*GFP^+^ population in comparison to untreated cells. (A,C) Nicotinamide added between days 7 and 14 did not affect the population of *Sox1*GFP-expressing cells. (D) Histogram showing the proportion of cells with different levels of *Sox1*GFP expression. Larger numbers of cells displayed weak *Sox1*GFP expression in cultures that were exposed to 10 mM nicotinamide between days 0 and 7 (red bars), compared with controls. (E-F) Addition of nicotinamide increased the population of GABAergic neurons from mESCs. Nicotinamide (10 mM) added to media generated a significant increase in the percentage of the total GABAergic neuron population at day 14, compared against the control untreated group. Some cells showed intense GABA immunoreactivity (yellow arrows) and some less strong GABA expression (white arrow). Scale bar 50 μm applies to all high magnification images. ***p<0.001, **p<0.01.

The effect of nicotinamide on monolayer-derived *Sox1*GFP-expressing neural progenitors (NPCs) was also assessed by quantifying GFP^+^ cells. Application of 10 mM nicotinamide between days 0 and 7 significantly reduced the percentage of GFP-expressing NPCs by day 14 of differentiation (unpaired t test, t = 5.6; *p<*0.001; 12.8 ± 1.8% vs. 30.5 ± 2.6% in controls; Fig 4(A) and 4(B)). No significant differences in the percentage of GFP^+^ cells were found between control and treatment conditions when nicotinamide was added at late differentiation stages (unpaired t test, t = 0.8; n.s.; 32.5 ± 2.8% vs. 36.2 ± 3.7% in controls; Fig 4(A)–4(C)).

Within monolayer cultures, variations in the intensity of GFP expression was evident, resulting in a spectrum from bright to low intensity GFP^+^ cells. It was hypothesised that cells expressing higher levels of GFP represented true *Sox1*^+^ NPCs, and weaker GFP^+^ cells were likely to be immature neuronal and glial cells that had down-regulated GFP expression, but still possessed GFP protein, yet to be degraded. This is likely due to the longer half-life of the GFP protein (~ 26 h), in contrast to the*Sox1* gene [24]. Therefore, to investigate the effect of nicotinamide on intense and weak *Sox1*GFP^+^ neural progenitor populations, GFP^+^ cells were scored for levels of reporter protein using OD measures. Data for OD values highlighted a marked reduction in the relative levels of GFP expression in cells treated with 10 mM nicotinamide between days 0 and 7, compared to untreated cultures. Nicotinamide applied for the first 7 days of differentiation almost completely eradicated “strong” *Sox1*GFP-expressing cells (i.e. true *Sox1*^+^ NPCs) from cultures (ANOVA: F(12,36) = 17.18, p<0.0001). Significantly more cells treated with nicotinamide from day 0–7 expressed low levels of *Sox1*GFP (0.2 units fluorescence) compared with control cultures (0 mM nicotinamide applied at days 0–7 or 7–14; (Tukey’s multiple comparison t-tests: P<0.05, Fig 4(D)). Nicotinamide did not induce significant differences in the relative levels of GFP expression in cells treated between days 7 and 14, further indicating that its action is differentiation stage-dependent.

In addition to neuronal differentiation, cultures were assessed for glial differentiation using the antibodies: glial fibrillary acidic protein (GFAP) and neural/glial antigen 2 (NG2). Nicotinamide had no effect on the numbers of glial cells produced, at day 14 (Supplementary S1 Fig). GFAP-expressing cells exhibiting long cellular projections similar to those of radial glial cells, were detected at day 14, albeit in small numbers, in striking contrast with the number of βIII-tubulin–expressing neurons generated at this time point. There was no significant effect on the percentage of GFAP+ cells at day 14 in cultures of cells incubated with nicotinamide between days 0 and 7 compared to control cultures, (unpaired t test, t = 1.0, n.s.) (0.3 ± 0.1% in nicotinamide treated cultures vs. 0.5 ± 0.2% in control conditions; Supplementary S1b Fig). Similarly, there was no difference in NG2 expression when comparing differentiated cells treated with or without nicotinamide (unpaired t test, t = 1.2, n.s.)(3.2 ± 0.5% in nicotinamide treated cultures vs. 2.3 ± 0.6% in control conditions; Supplementary S1c Fig). The morphology of glial cells in the nicotinamide-treated cultures appeared normal, as expected in a mixed mature neural culture.

These important data indicate that nicotinamide administration at the initial stages of differentiation rapidly decreased the *Sox1*GFP^+^ NPC population, driving the neural progenitors to become further differentiated to mature neurons, but had no influence on glial differentiation.

### Nicotinamide treatment enhanced the production of GABAergic neurons from mESCs

Double immunofluorescence with anti-GABA and neuron-specific βIII-tubulin demonstrated that the majority of *Sox1*GFP-derived neurons at day 14 in culture were immunopositive for GABA, in line with other studies using the *Sox1*GFP knock-in 46C cell line [21,24].Therefore, consistent with the higher proportion of βIII-tubulin neurons observed in nicotinamide treated groups, 10 mM nicotinamide more than doubled the percentage of GABAergic neurons of the total cell population labelled with DAPI, compared to un-treated cells (unpaired t test, t = 8.5; *p<*0.001; 22.8 ± 1.2% vs. 10.7 ± 0.8%; Fig 4(E) and 4(F)). These data demonstrate that *Sox1*GFP derived-mESCs differentiated with an early exposure to nicotinamide exhibit higher yields of GABAergic neurons per culture.

### Nicotinamide differentiation accelerated neuronal maturation in GABAergic populations

The effect of nicotinamide on neurodevelopment and maturation was next investigated in *Sox1*GFP mESC-derived GABAergic neuronal populations. Early neuronal development commences with elongation of neurite processes followed by axon differentiation, dendritic branching and synaptic formation. In this context, several types of GABAergic neuronal morphologies were observed in monolayer cultures at day 14 of differentiation, i.e. GABAergic neurons with short, medium and long neurite processes. Therefore, we assessed the effect of nicotinamide on primary neurite outgrowth (i.e. length of the longest neurite), total neurite outgrowth and the number of primary neurite branches per neuron.

**Fig 5:**
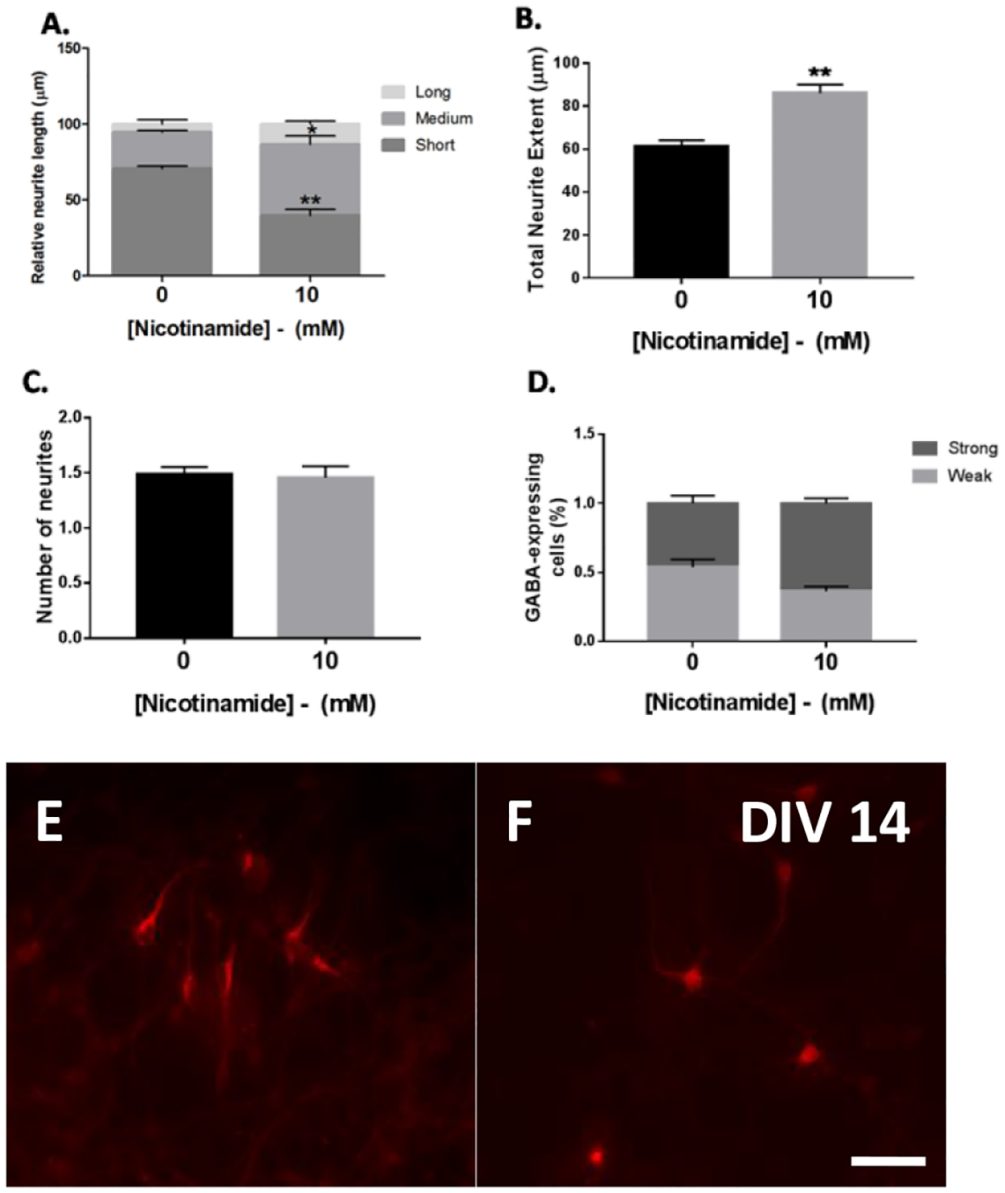
Nicotinamide enhanced neuronal maturation in GABAergic populations. (A and E) The proportion of 46C-derived cells displaying “short” primary processes was significantly decreased in cultures exposed to 10 mM nicotinamide between days 0 and 7, concomitant with an obvious increased trend for “longer” neurite processes, compared with controls (F). (B) Total length of neurites was significantly increased in the presence of nicotinamide. (C) There was no difference in the number of primary neurites per neurons between controls and nicotinamide-stimulated conditions. (D) An upward trend in the proportion of mESC-derived cells displaying “strong” levels of GABA expression was demonstrated in cultures exposed to 10 mM nicotinamide, which correlated with a downward trend in “weaker”-expressing GABAergic cells, compared with control conditions. ***p<0.001, **p<0.01, *p<0.05. Micrographs show morphologies of GABA+ neurons differentiated in control conditions (E), or cultures treated with 10 mM nicotinamide (F). Scale bar = 100 μm.

In cultures treated with 10 mM nicotinamide, there was an upward trend in the proportion of cells exhibiting “medium” (51.5 ± 1.3 vs. 47.8 ± 4.6 in controls; n.s.) and “long” (34.5 ± 2.9 vs. 15.2 ± 1.2 in controls; n.s.) neurite outgrowths, and therefore a significant reduction of the proportion of GABAergic neurons displaying “short” primary neurite branches (14.0 ± 2.4 vs. 36.9 ± 4.2 in control conditions; p<0.001; Fig 5(A), 5(E) and 5(F)). Total neurite extent was significantly increased in 10 mM nicotinamide cultures, compared to controls (unpaired t test, t = 5.0; p<0.01; 86.05μm ± 4.0 vs. 61.11μm ± 3.0 in control groups; Fig 5(B)). Nicotinamide treatment induced no significant changes in the number of primary neurites per neuron, compared to untreated conditions (unpaired t test, t = 0.2; n.s.; 1.5 ± 0.1 vs. 1.5 ± 0.1 in control groups; Fig 5(C)). Overall, these data suggest that nicotinamide was functioning to exert a neurite growth-promoting action on young neuronal subtypes.

Further, GABAergic-expressing neurons exhibited differences in immunofluorescence intensities post-staining with a primary antibody against GABA in monolayer cultures, resulting in a spectrum from weak to strong fluorescence. Cells expressing higher levels of immunostaining are likely to represent more mature GABAergic neuronal subtypes, since strong staining reflects more neurotransmitter content [25]. Therefore, to further investigate the effect of nicotinamide on the maturation of GABAergic neurons, individual cell bodies were scored for levels of immunofluorescence using OD measures and cells were categorised as either ‘weak’ (OD ≤ 0.1 units) or ‘strong’ (OD ≥ 0.2 units) expressers.

In agreement with the rapid maturation effect on neurite outgrowth, we observed a substantial increase in the proportion of GABAergic cells displaying higher neurotransmitter content within neuronal cell bodies. Analysis indicated an upward trend in the percentage of neurons expressing intense GABA immunoreactivity, in cultures differentiated with 10 mM nicotinamide (0.6 ± 0.0 vs. 0.5 ± 0.1 in control conditions; Fig 5(D)). Correspondingly, there was a downward trend in the percentage of weak-expressing GABAergic neurons (0.4 ± 0.0 vs. 0.5 ± 0.1 in control conditions; Fig 5(D)).

Thus, *Sox1*GFP-derived neurons differentiated with nicotinamide between days 0 and 7 underwent neuronal morphogenesis earlier than cells induced with N2B27 medium alone. These findings are consistent with the accelerated differentiation of mESCs to mature neurons in the presence of nicotinamide.

## Discussion

This novel study reports that nicotinamide is sufficient to induce accelerated neural specification from mESCs which further progressed to form GABAergic neuronal populations with enhanced efficiency and maturity. Results obtained in this study show fundamental effects of nicotinamide during the initial phases of ESC development, rather than at later stages of neuronal differentiation [26]. Within 2 days of culture, nicotinamide had dramatically reduced the number of Oct4-expressing pluripotent colonies and increased GFP fluorescence, thereby suggesting that nicotinamide accelerated the conversion of mESCs to a neuroectoderm fate. Previous findings reported by Griffin et al. demonstrated that treatment of mESC (46C *Sox1*GFP reporter cell line) monolayer cultures with nicotinamide at the early onset of development decreased the total number of cells in cultures, resulting in a greater ratio of neurons to non-neuronal cells [26]. Specifically, by day 14 in this study, nicotinamide treatment (from day 0–7) reduced the populations of undifferentiated cells and Sox1GFP+ progenitors, in addition to reducing Oct4 and GFP expression in the cultures concomitant with enhanced expression of βIII-tubulin, indicative of accelerated neuronal differentiation. Thus, this marked reduction in distinct cell populations forms a mutually supportive body of data compatible with the previous data showing reduction of the total cell population at this time point. The cellular mechanisms of nicotinamide in this regard are currently unknown. Therefore, further studies are required to determine whether nicotinamide promotes inhibition of proliferation, and/or whether nicotinamide induces cell death in specific cell populations. In this context, Vaca et al found that nicotinamide could cause an 80% reduction in ESC proliferation [12], supporting the former mechanism of action, whilst Idelson et al found that prevention of apoptosis in neural populations, rather than augmentation of their proliferation resulted in enhanced RPE differentiation [15]. It is interesting to note that addition of nicotinamide from day 0 of monolayer differentiation elicited a more potent effect on neuronal differentiation by day 7, which promoted significantly enhanced yields of βIII-tubulin^+^ neurons. In contrast, nicotinamide induced a less marked effect on neural induction between days 2 and 7, implying that the inductive role of nicotinamide may function during a narrow time window prior to or at the stage of *Sox1* expression during mESC differentiation. This data is in line with recent observations that increased nicotinamide-N-methyl transferase (NNMT) activity may retain embryonic stem cells in their naïve state, through regulation of epigenetic events, by creating low levels of NNMT’s substrates S-adenosylmethionine and nicotinamide, leading to a subsequent reduction in histone methylation [27]. Furthermore, this early and robust effect suggests that nicotinamide may be acting at the initial stages of ESC differentiation to influence the transition of mESCs into neuroectoderm or indeed driving a switch from neural progenitors to neurons in the differentiating cells. Similarly, the vitamin A derivative, RA has been shown to regulate the proliferation-to-differentiation switch [28]. Specifically, RA has been reported to promote differentiation and inhibit cell proliferation by directing cell cycle arrest in ESCs. With particular relevance to the findings discussed in this study, early administration of RA to differentiating adherent mono-cultures was found to restrict the proliferation of Sox1+ neuroectodermal precursors, resulting in enhanced neurite outgrowth and βIII-tubulin expression. In the present study, nicotinamide significantly reduced the percentage of GFP+ cells, concomitant with a decrease in the number of actively proliferating *Sox1*GFP+ NPC cells. As far as we are aware, this is the first report to demonstrate that the vitamin B_3_ metabolite may be influencing a similar switch to RA—that is a neural proliferation-to-differentiation switch from neural progenitors to neurons.

In this study, it is therefore reasonable to hypothesise that the initial higher frequency of *Sox1*GFP^+^neural progenitors in monolayer cultures may be due to enhanced viability, or conversion of ESCs to neural progenitor cells during the early stages of development. In this regard, one study showed that prevention of apoptosis was one of the mechanisms mediating the promotion of neural specification by nicotinamide. Nicotinamide was shown to reduce cell death of human ESC-derived NPCs upon neural induction, via inhibition of PARP, thereby enhancing neutralization [29]. However, it is important to note that higher doses of nicotinamide (20 mM) induced cytotoxic effects on cells within 3 days of application [26]. This is in agreement with other differentiation studies that also found that high doses of the active metabolites of vitamin A (retinoic acid: 10^−6^μM) and vitamin C (ascorbic acid: 500–5000 μM) had obvious negative effects on cell survival [8,23]. In this context, review papers published by Williams *et al* propose a hypothetical link between a hypervitaminosis B_3_ state and modern disease phenomena, resulting from a modern Western diet abundant in meat and vitamin supplements [30,31]. Excess nicotinamide has been linked with lower levels of dopamine when administered postnatally to mice (potentially through Sirt1 inhibition) [32]; and neurodegeneration in the Parkinsonian brain [33]. Conversely, some doses of nicotinamide have been shown to be broadly neuroprotective in mouse models of MPTP-induced Parkinson’s [34], implying that levels of this vitamin require tight regulation in order to support and sustain normal neuronal functioning.

By day 14 of monolayer differentiation, early nicotinamide treatment (from day 0–7) had reduced both Oct4 and GFP expression in the cultured cells. Two quantitative methods of pluripotency: quantification of Oct4 immunoreactive colonies; and measurement of Oct4-labelled areas within DAPI stained colonies, both revealed a significantly reduced Oct4^+^ pluripotent stem cell population in cultures at day 14. Nicotinamide supplementation also reduced the *Sox1*GFP^+^ NPC numbers at day 14 of the monolayer protocol. Taken with previous findings that nicotinamide increases the number of ßIII tubulin^+^ neurons at day 14 [26], itis therefore reasonable to conclude that, in the presence of nicotinamide, more NPCs differentiated into neurons earlier. Interestingly, the majority of cells treated with nicotinamide showed uniformly weaker GFP expression. Thus, our findings are in support of previous evidence where nicotinamide has been shown to eliminate Oct4^+^ dividing cells and progenitor cells from pluripotent stem cell cultures, thereby speeding up differentiation into clinically relevant lineages [15–17]. Data reveal that the GFAP protein and the early OPC marker, NG2, were expressed in very few cells of the Sox1GFP-derived cultures at day 14; constituting less than 5% of the total cell population. In addition, mature GFAP+ cells displaying characteristic large flattened cell bodies were absent from monolayer cultures at this time-point. Thus, it is reasonable to conclude that day 14 of monolayer differentiation might be too early to assess for the effect of nicotinamide on glial fate, in line with previous reports in the literature.

It has been reported that neural progenitor formation and neuronal fate specification are two closely linked processes occurring during ESC differentiation, which may be instructed by the same signals [35]. GABAergic neurons are of particular interest for cell transplantation due to their degeneration in the neostriatum which underlies the neuropathology of HD. In this study, under control conditions, the vast majority of neuronal cells produced by monolayer culture were GABA^+^, reflecting similar findings in other studies using *Sox1*GFP-derived cultures [21,24,36]. Results reveal that nicotinamide treatment of mESCs led to a significant increase in the proportion of the total cell population that were GABAergic neurons, highlighting the potential of nicotinamide as a useful candidate in future protocols for generating GABAergic populations.

Several reports support a role for nicotinamide in cellular maturation and differentiation processes [37,38].In this study, nicotinamide treatment led to enhanced neurite elongation and a tendency towards increased levels of neurotransmitter content within the cell bodies of GABA-expressing neuronal subpopulations, therefore indicative of an accelerative effect on the maturation of mESC-derived neurons. We did not observe any aberrant cell phenotypes in nicotinamide-treated cultures, suggesting that there are no untoward effects of nicotinamide, i.e. the cells appear to be normal, and equivalent of a more mature neuronal culture. These important findings provide crucial evidence that under serum-free and factor-free conditions, enhanced neural subtype commitment and neuronal morphogenesis were predominantly due to the independent effect of nicotinamide, applied during the early stages of stem cell differentiation (i.e. day 0–7). These findings strongly suggest that nicotinamide could be applied to current neuronal protocols during the early stages of neural conversion, in combination with factors known to enhance neuronal phenotypes and to promote subtype specification. In this regard, to fully determine the effects of nicotinamide on GABA differentiation, future work should focus on the effects of nicotinamide in conjunction with ventralising factors using Shh or an agonist, purmorphamine, which are necessary to promote the emergence of a forebrain phenotype from ESCs [39], or indeed with RA which has been shown to function as a key GABAergic differentiation signal in the basal ganglia circuitry [40].

Currently, the challenge in ESC biology to advance the potential of pluripotent stem cell sources is to establish criteria for determining effective differentiation. Thus our work is highly relevant to translate effective laboratory cell-based protocols to patients in clinical trials, opening up the possibility of applying nicotinamide as a differentiation agent to aid the conversion of stem cells to GABAergic neurons, helping to increase the efficiency of this process and the safety of the cells produced.

## Methods

All reagents used in this study were from Sigma, UK unless specified otherwise.

### Embryonic stem cell culture

The mESC line 46C (a kind gift of Professor Meng Li, Neuroscience and Mental Health Research Institute, Cardiff University, UK) carrying a green fluorescent protein knock-in reporter targeted to the *Sox1* promoter (transiently expressed during the neural progenitor stage) was used throughout this study. mESCs were cultured in Glasgow Modified Eagles Medium (Invitrogen, UK) with the addition of 10% FCS, 0.1 M β-mercaptoethanol, 1 mM L-glutamine, 10 mM non-essential amino acids, 100 mM sodium pyruvate and 100 U/ml leukaemia inhibitory factor. Undifferentiated cells were routinely passaged every two days re-plating at a density of 1 x 10^6^ cells/cm^2^. mESCs were maintained on 0.1% gelatin-coated tissue culture plastic at 37°C in a 5% CO_2_ incubator.

### Neural differentiation

Monolayer neural differentiation was based on a previous protocol using the 46C line established by Ying *et al* [21]. Briefly, for neural induction, undifferentiated mESCs were plated at a density of 9 x 10^4^ cells/cm^2^ per well of a 0.1% gelatin-coated 6-well dish (day 0). N2B27 serum-free medium (5 ml) consisting of a 1:1 ratio of DMEM/F12 (Invitrogen) and Neurobasal media (Invitrogen) containing 0.5% N2 (Fisher Scientific, Loughborough, UK), 1% B27 (Fisher Scientific), 200mM _L_-glutamine and 0.1M β-mercaptoethanol was added to each well. The medium was refreshed every alternate day. On day 7, single cells (3 x 10^4^) were replated in 30 μl N2B27 medium on 13 mm glass coverslips (Fisher Scientific) pre-treated with poly-L-lysine (PLL) (10 μg/ml) and laminin (2 μg/ml) in 24-well plates. After 4–6 h of incubation, the wells were flooded with 500 μl of N2B27 medium, which was refreshed every other day until day 14. During this 14-day period, cells were treated with the biologically active metabolite nicotinamide (10 mM), added to the base medium between either days 0 and 7 or days 7 and 14. Two additional culture periods with nicotinamide were also investigated: day 0 to 2 and day 2 to 7. Control groups were not treated with nicotinamide.

### Cell sample analysis

To perform a comprehensive time-course analysis of the effect of nicotinamide on the progression of mESCs through neural induction and neuronal differentiation, immunocytochemistry using fluorescent antibodies was carried out (Fig 1). Quantitative analysis techniques included cell counts, optical density (OD) measures and area measurements for pluripotent colonies.

### Immunofluorescence staining

Differentiated cells were fixed with 4% paraformaldehyde (PFA) for 20 min at 4°C. Fixed cells were washed three times with Tris buffered saline (TBS) for 5 min. Cells were blocked against non-specific binding and permeabilised with 0.02% Triton X-100 and 5% normal goat serum (NGS) (PAA, The Cell Culture Company, Somerset, UK), for 1 h at room temperature (RT). Primary antibodies diluted in 1% NGS blocking buffer were added to cultured cells overnight at 4°C. Negative primary controls consisted of cells treated with blocking buffer without the addition of primary antibodies. The next day, three TBS washes were applied to the cells for 5 min followed by incubation with fluorescent dye-conjugated secondary antibodies, diluted to 1:300 in 1% NGS blocking buffer, for 2 h at RT. Primary and secondary antibodies and dilutions are reported in S1 Table. Cultures were washed three times with TBS for 5 min. Coverslips were mounted onto microscope slides using Vectashield hardset mounting medium containing 4', 6-diamidino-2-phenylindole (DAPI) (Vector Labs, Peterborough, UK) to counterstain cell nuclei, before visualisation the following day.

### Cell quantification

Cell samples were visualised using fluorescence microscopy (Nikon Eclipse 80i microscope; Nikon UK Limited, Kingston upon Thames, UK) and images acquired using a Hamamatsu ORCA camera (Hamamatsu Photonics UK Limited, Welwyn Garden City, UK). NIS-Elements imaging software, version BR 3.2 (Nikon UK Limited) was used to manually quantify positive antibody labelling in DAPI-stained cultures. Independent experiments were replicated three times and three to four coverslips per group were counted within an experiment. Specific cell populations were analysed by capturing six to eight random fields per coverslip. Data obtained represent an average of approximately 50 DAPI-labelled cells per field of view. Clusters containing dense populations of neural progenitor and neuronal cells were excluded from data collection, since these cell networks were not countable.

### Cell morphology analysis

Morphometric analyses were conducted to assess the extent of neuronal differentiation in the presence of nicotinamide. Neuronal cells were photographed from eight to ten random fields per coverslip from three independent experiments using a high power objective lens (i.e. x 40 lens). Neuronal cells within cell clusters were omitted from morphometric analysis. Four morphological parameters were investigated: (1) measurement of neurotransmitter content within neuronal cell bodies (soma) using OD measures; (2) number of primary neurite branches; (3) length of longest neurite (μm), and (4) total neurite extent (μm). Neuronal processes greater than two cell diameters in length were considered as true neurites and neurites of labelled cells were manually traced using the ImageJ plug-in NeuronJ (version 1.4.2; NIF). Neurite length was defined as the distance from the soma to the tip of the longest primary neurite and the combined lengths of all neurites per cell were defined as total neurite length.

### Optical density

OD measures were obtained to determine levels of protein expression of specific neuronal populations derived from *Sox1*GFP mESCs, using ImageJ image analysis software (version 1.45s; NIH). Cell samples were captured at fixed exposure settings using a Hamamatsu ORCA camera with NIS Elements imaging software. Cells located within clusters were excluded from OD analyses. OD values were evaluated by converting each colour image to grayscale and calibrated using an optical density step tablet. OD readings were then corrected for the background.

### Statistical analysis

Statistical analysis was performed using GraphPad Prism version 5.00 (GraphPad Software Inc., La Jolla, California, USA). Data plotted on graphs is expressed as mean ± standard error of the mean (SEM). An unpaired two-tailed t test was used to compare two sets of data. A level of *p*<0.05 was used as a limit for statistical significance.

## Supporting information

S1 Fig Treatment of adherent cultures with nicotinamide elicited no effect on glial differentiation. (A) Immunofluorescence images of glial populations generated from mESC-derived monolayer cultures, showing immunostaining with antibodies specific to the astrocyte marker, GFAP and the OPC marker, NG2. GFAP-expressing cells with leading processes and elongated bodies were observed at day 14 of monolayer differentiation, and NG2-positive cells possessed multiple processes, representative of their morphology *in vivo*. Cell nuclei counterstained with DAPI (blue). Scale bar = 50 μm. (B) GFAP+ and (C) NG2+ cells were detected in cultures at a very low percentage. No significant effects on glial expression were observed following treatment of cultures with 10 mM nicotinamide between days 0–7.
(TIF)

S1 Table List of antibodies used for immunofluorescence studies. (TIF)

## References

[1] Barker R. A., Drouin-Ouellet J. & Parmar M. Cell-based therapies for Parkinson disease—past insights and future potential. *Nat*. *Rev*. *Neurol*.11, 492–503 (2015). doi: 10.1038/nrneurol.2015.123 26240036 10.1038/nrneurol.2015.123

[2] Avior Y., Sagi I. & Benvenisty N. Pluripotent stem cells in disease modelling and drug discovery. *Nat*. *Rev*. *Mol*. *Cell Biol*. (2016). doi: 10.1038/nrm.2015.27 26818440 10.1038/nrm.2015.27

[3] Dunnett S. B. & Rosser A. E. Challenges for taking primary and stem cells into clinical neurotransplantation trials for neurodegenerative disease. *Neurobiol*. *Dis*.61, 79–89 (2014). doi: 10.1016/j.nbd.2013.05.004 23688854 10.1016/j.nbd.2013.05.004

[4] Blumberg B. An essential role for retinoid signaling in anteroposterior neural specification and neuronal differentiation. *Semin*. *Cell Dev*. *Biol*.8, 417–28 (1997). doi: 10.1006/scdb.1997.0165 15001080 10.1006/scdb.1997.0165

[5] Papalopulu N. & Kintner C. A posteriorising factor, retinoic acid, reveals that anteroposterior patterning controls the timing of neuronal differentiation in Xenopus neuroectoderm. *Development* 122, 3409–18 (1996). 8951057 10.1242/dev.122.11.3409

[6] Orme R. P., Gates MA & Fricker-Gates RA. A multiplexed quantitative proteomics approach for investigating protein expression in the developing central nervous system. *J*. *Neurosci*. *Methods* 191, 75–82 (2010). doi: 10.1016/j.jneumeth.2010.06.009 20558204 10.1016/j.jneumeth.2010.06.009

[7] Orme R. P., Bhangal M. S. & Fricker RA. Calcitriol imparts neuroprotection in vitro to midbrain dopaminergic neurons by upregulating GDNF expression. *PLoS One* 8, e62040 (2013). doi: 10.1371/journal.pone.0062040 23626767 10.1371/journal.pone.0062040 PMC3633905

[8] Bagga V., Dunnett S. B. & Fricker-Gates RA. Ascorbic Acid Increases the Number of Dopamine Neurons In Vitro and in Transplants to the 6-OHDA-Lesioned Rat Brain. *Cell Transplant*.17, 763–773 (2008). 19044203 10.3727/096368908786516774

[9] Yan J., Studer L. & McKay R. D. Ascorbic acid increases the yield of dopaminergic neurons derived from basic fibroblast growth factor expanded mesencephalic precursors. *J*. *Neurochem*.76, 307–11 (2001). 11146004 10.1046/j.1471-4159.2001.00073.x

[10] Shin E., Palmer M. J., Li M. & Fricker R. A. GABAergic neurons from mouse embryonic stem cells possess functional properties of striatal neurons in vitro, and develop into striatal neurons in vivo in a mouse model of Huntington’s disease. *Stem Cell Rev*.8, 513–31 (2012). doi: 10.1007/s12015-011-9290-2 21720791 10.1007/s12015-011-9290-2

[11] Engberg N., Kahn M., Petersen D. R., Hansson M. & Serup P. Retinoic acid synthesis promotes development of neural progenitors from mouse embryonic stem cells by suppressing endogenous, Wnt-dependent nodal signaling. *Stem Cells* 28, 1498–1509 (2010). doi: 10.1002/stem.479 20665854 10.1002/stem.479

[12] Vaca P. et al. Nicotinamide induces differentiation of embryonic stem cells into insulin-secreting cells. *Exp*. *Cell Res*.314, 969–974 (2008). doi: 10.1016/j.yexcr.2007.11.019 18234191 10.1016/j.yexcr.2007.11.019

[13] Vaca P., Berná G., Martín F. & Soria B. Nicotinamide induces both proliferation and differentiation of embryonic stem cells into insulin-producing cells. *Transplant*. *Proc*.35, 2021–2023 (2003). 12962883 10.1016/s0041-1345(03)00735-8

[14] Zhang Y., Wang J., Chen G., Fan D. & Deng M. Inhibition of Sirt1 promotes neural progenitors toward motoneuron differentiation from human embryonic stem cells. *Biochem*. *Biophys*. *Res*. *Commun*.404, 610–4 (2011). doi: 10.1016/j.bbrc.2010.12.014 21144831 10.1016/j.bbrc.2010.12.014

[15] Idelson M. et al. Directed Differentiation of Human Embryonic Stem Cells into Functional Retinal Pigment Epithelium Cells. *Cell Stem Cell* 5, 396–408 (2009). doi: 10.1016/j.stem.2009.07.002 19796620 10.1016/j.stem.2009.07.002

[16] Buchholz D. E. et al. Rapid and Efficient Directed Differentiation of Human Pluripotent Stem Cells Into Retinal Pigmented Epithelium. *Stem Cells Transl*. *Med*.2, 384–393 (2013). doi: 10.5966/sctm.2012-0163 23599499 10.5966/sctm.2012-0163 PMC3667566

[17] Parsons X. H. et al. Efficient derivation of human cardiac precursors and cardiomyocytes from pluripotent human embryonic stem cells with small molecule induction. *J*. *Vis*. *Exp*. e3274 (2011). doi: 10.3791/3274 22083019 10.3791/3274 PMC3308594

[18] Caplan A. I. & Ordahl C. P. Irreversible gene repression model for control of development. *Science* 201, 120–30 (1978). 351805 10.1126/science.351805

[19] Fu L., Doreswamy V. & Prakash R. The biochemical pathways of central nervous system neural degeneration in niacin deficiency. *Neural Regen*. *Res*.9, 1509–13 (2014). doi: 10.4103/1673-5374.139475 25317166 10.4103/1673-5374.139475 PMC4192966

[20] Williams A. & Ramsden D. Nicotinamide: A double edged sword. *Park*. *Relat*. *Disord*.11, 413–420 (2005). 10.1016/j.parkreldis.2005.05.011 16183323

[21] Ying Q.-L., Stavridis M., Griffiths D., Li M. & Smith A. Conversion of embryonic stem cells into neuroectodermal precursors in adherent monoculture. *Nat*. *Biotechnol*.21, 183–186 (2003). doi: 10.1038/nbt780 12524553 10.1038/nbt780

[22] Lowell S., Benchoua A., Heavey B. & Smith A. G. Notch promotes neural lineage entry by pluripotent embryonic stem cells. *PLoS Biol*.4, 805–818 (2006). 10.1371/journal.pbio.0040121 PMC1431581 16594731

[23] Lu J. et al. All-trans retinoic acid promotes neural lineage entry by pluripotent embryonic stem cells via multiple pathways. *BMC Cell Biol*.10, 57 (2009). doi: 10.1186/1471-2121-10-57 19642999 10.1186/1471-2121-10-57 PMC2728515

[24] Chung S. et al. Genetic selection of sox1GFP-expressing neural precursors removes residual tumorigenic pluripotent stem cells and attenuates tumor formation after transplantation. *J*. *Neurochem*.97, 1467–1480 (2006). doi: 10.1111/j.1471-4159.2006.03841.x 16696855 10.1111/j.1471-4159.2006.03841.x PMC2610439

[25] Shin E., Forsyth N. R. & Fricker RA. The effect of physiological oxygen levels on GABAergic neuronal differentiation from mouse embryonic stem cells. *Stem Cell Stud*.2, 13–20 (2012).

[26] Griffin S. M., Pickard M. R., Orme R. P., Hawkins C. P. & Fricker R. A. Nicotinamide promotes neuronal differentiation of mouse embryonic stem cells in vitro. *Neuroreport* 24, 1041–6 (2013). doi: 10.1097/WNR.0000000000000071 24257250 10.1097/WNR.0000000000000071

[27] Sperber H. et al. The metabolome regulates the epigenetic landscape during naive-to-primed human embryonic stem cell transition. *Nat*. *Cell Biol*.17, 1523–35 (2015). doi: 10.1038/ncb3264 26571212 10.1038/ncb3264 PMC4662931

[28] Janesick A., Wu S. C. & Blumberg B. Retinoic acid signaling and neuronal differentiation. *Cell*. *Mol*. *Life Sci*.72, 1559–1576 (2015). doi: 10.1007/s00018-014-1815-9 25558812 10.1007/s00018-014-1815-9 PMC11113123

[29] Cimadamore F. et al. Nicotinamide rescues human embryonic stem cell-derived neuroectoderm from parthanatic cell death. *Stem Cells* 27, 1772–81 (2009). doi: 10.1002/stem.107 19544437 10.1002/stem.107 PMC4151857

[30] Williams A. C. & Dunbar R. I. M. Big brains, meat, tuberculosis, and the nicotinamide switches: co-evolutionary relationships with modern repercussions? *Int*. *J*. *Tryptophan Res*.6, 73–88 (2013). doi: 10.4137/IJTR.S12838 24250227 10.4137/IJTR.S12838 PMC3825668

[31] Williams A. C., Cartwright L. S. & Ramsden D. B. Parkinson’s disease: the first common neurological disease due to auto-intoxication? QJM 98, 215–26 (2005). doi: 10.1093/qjmed/hci027 15728403 10.1093/qjmed/hci027

[32] Lee J.-Y. et al. Nicotinamide reduces dopamine in postnatal hypothalamus and causes dopamine-deficient phenotype. *Neurosci*. *Lett*.461, 163–6 (2009). doi: 10.1016/j.neulet.2009.06.005 19539713 10.1016/j.neulet.2009.06.005

[33] Parsons R. B., Smith S. W., Waring R. H., Williams A. C. & Ramsden D. B. High expression of nicotinamide N-methyltransferase in patients with idiopathic Parkinson’s disease. *Neurosci*. *Lett*.342, 13–16 (2003). 12727306 10.1016/s0304-3940(03)00218-0

[34] Anderson D. W., Bradbury K. A. & Schneider J. S. Broad neuroprotective profile of nicotinamide in different mouse models of MPTP-induced parkinsonism. *Eur*. *J*. *Neurosci*.28, 610–7 (2008). doi: 10.1111/j.1460-9568.2008.06356.x 18702732 10.1111/j.1460-9568.2008.06356.x

[35] Parmar M. & Li M. Early specification of dopaminergic phenotype during ES cell differentiation. *BMC Dev*. *Biol*.7, 86 (2007). doi: 10.1186/1471-213X-7-86 17640353 10.1186/1471-213X-7-86 PMC1978208

[36] Nefzger C. M. et al. Lmx1a allows context-specific isolation of progenitors of GABAergic or dopaminergic neurons during neural differentiation of embryonic stem cells. *Stem Cells* 30, 1349–1361 (2012). doi: 10.1002/stem.1105 22495882 10.1002/stem.1105

[37] Miura M. & Kameda Y. Nicotinamide promotes long-term survival and extensive neurite outgrowth in ultimobranchial C cells cultured from chick embryos. *J*. *Comp*. *Neurol*.492, 334–48 (2005). doi: 10.1002/cne.20731 16217794 10.1002/cne.20731

[38] Giammona L. M. et al. Mechanistic studies on the effects of nicotinamide on megakaryocytic polyploidization and the roles of NAD+ levels and SIRT inhibition. *Exp*. *Hematol*.37, 1340–1352.e3 (2009). doi: 10.1016/j.exphem.2009.08.004 19715739 10.1016/j.exphem.2009.08.004 PMC2763937

[39] Rosser A. & Svendsen C. N. Stem cells for cell replacement therapy: a therapeutic strategy for HD? *Mov*. *Disord*.29, 1446–54 (2014). doi: 10.1002/mds.26026 25216372 10.1002/mds.26026

[40] Chatzi C., Brade T. & Duester G. Retinoic acid functions as a key gabaergic differentiation signal in the basal ganglia. *PLoS Biol*.9, (2011). 10.1371/journal.pbio.1000609 PMC3075211 21532733

