# Supplementary figures and images for: Nicotinamide alone accelerates the conversion of mouse embryonic stem cells into mature neuronal populations
Source: PLoS One. 2017 Aug 17;12(8):e0183358. doi: 10.1371/journal.pone.0183358 (PMC5560552; doi:10.1371/journal.pone.0183358)

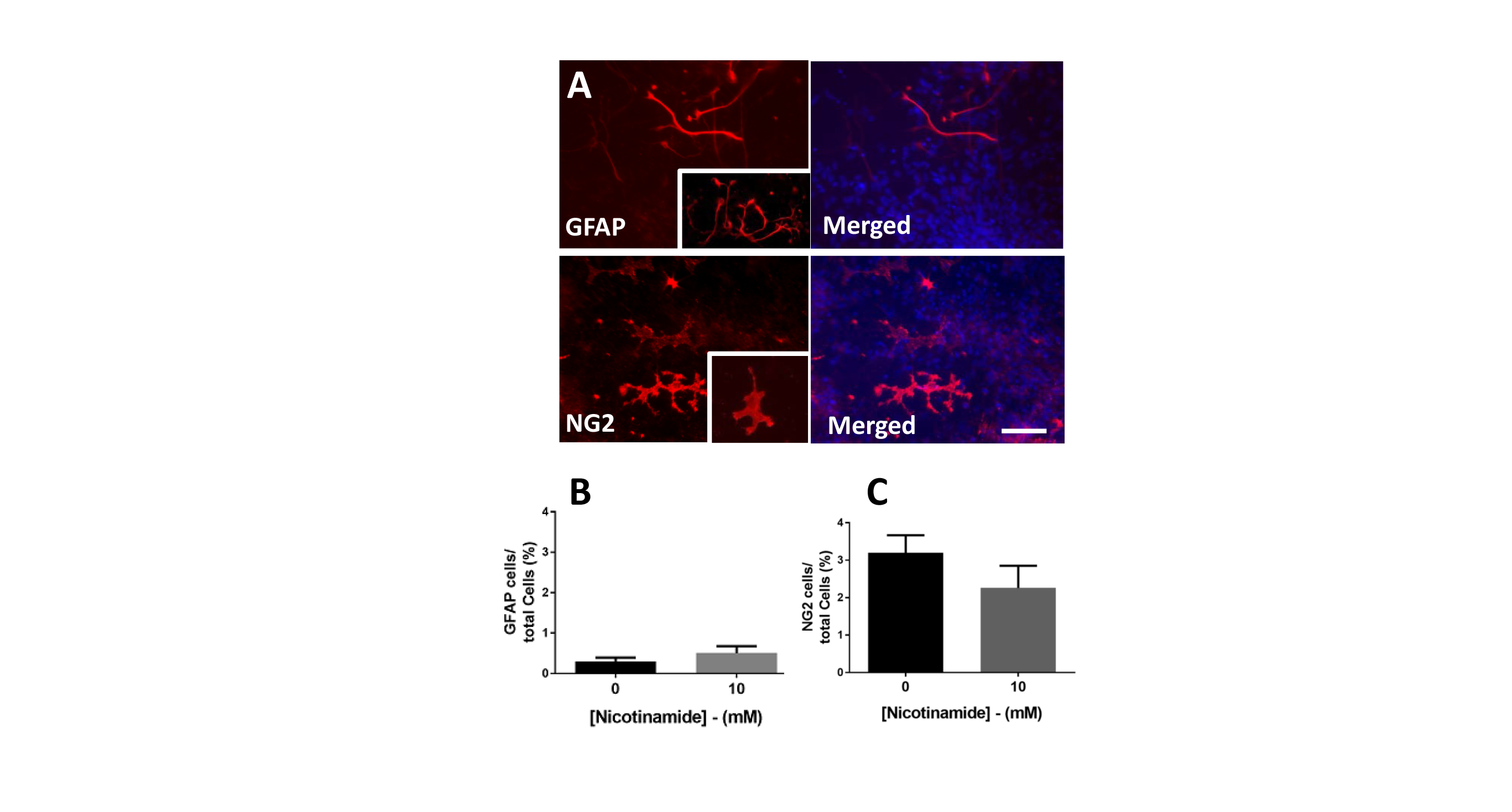

Supplement: S1 Fig — (A) Immunofluorescence images of glial populations generated from mESC-derived monolayer cultures, showing immunostaining with antibodies specific to the astrocyte marker, GFAP and the OPC marker, NG2. GFAP-expressing cells with leading processes and elongated bodies were observed at day 14 of monolayer differentiation, and NG2-positive cells possessed multiple processes, representative of their morphology in vivo. Cell nuclei counterstained with DAPI (blue). Scale bar = 50 μm. (B) GFAP+ and (C) NG2+ cells were detected in cultures at a very low percentage. No significant effects on glial expression were observed following treatment of cultures with 10 mM nicotinamide between days 0–7. (TIF) [file pone.0183358.s001.tif]

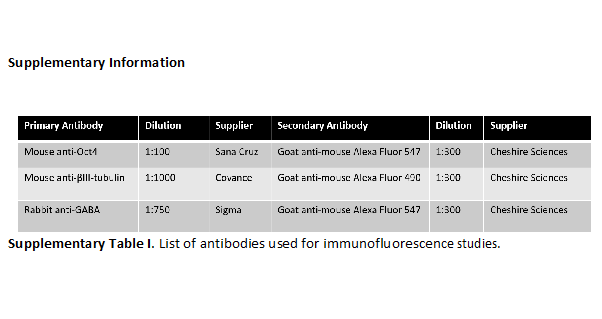

Supplement: S1 Table — (TIF) [file pone.0183358.s002.tif]
